# Sortase Enzyme-Mediated Generation of Site-Specifically Conjugated Antibody Drug Conjugates with High *In Vitro* and *In Vivo* Potency

**DOI:** 10.1371/journal.pone.0131177

**Published:** 2015-07-01

**Authors:** Roger R. Beerli, Tamara Hell, Anna S. Merkel, Ulf Grawunder

**Affiliations:** NBE-Therapeutics AG, Hochbergerstrasse, Basel, Switzerland; Baker IDI Heart and Diabetes Institute, AUSTRALIA

## Abstract

Antibody drug conjugates (ADCs) have recently been proven to be highly potent anti-tumor drugs, typically exceeding the efficacy of conventional monoclonal antibodies (mAbs). ADCs are currently produced by chemical conjugation of a small-molecule toxin to the mAb through lysine or cysteine side chains. This leads to heterogeneous mixtures of ADCs in which variable numbers of drugs are conjugated to individual antibodies and in which the site of conjugation cannot be defined. Consequently, there is currently significant interest in further development of drug conjugation technologies, with a particular focus on site-specific payload conjugation. Here, we present an enzymatic conjugation platform based on the *S*. *aureus* sortase A-mediated transpeptidation reaction, allowing the efficient generation of ADCs with toxins conjugated to pre-defined sites at pre-defined drug-to-antibody ratios. For this, two modifications were introduced: first, immunoglobulin heavy (IgH) and light (IgL) chains were modified at their C-termini by addition of the sortase A recognition motif LPETG, and second, the small molecule tubulin polymerization inhibitors monomethylauristatin E (MMAE) and maytansine were modified by addition of a pentaglycine peptide, thus making them suitable substrates for sortase A-mediated transpeptidation. We demonstrate efficient generation and characterization of the anti-CD30 ADC Ac10-vcPAB-MMAE, an enzymatically conjugated counterpart of brentuximab vedotin (Adcetris), as well as several anti-HER-2 ADCs including trastuzumab-maytansine, the counterpart of trastuzumab emtansine (Kadcyla). ADCs generated in this manner were found to display *in vitro* cell killing activities indistinguishable from the classic conjugates. Further, when tested *in vivo* in a HER-2-overexpressing ovarian cancer xenograft mouse model, enzymatically generated trastuzumab-maytansine was found to lead to complete regression of established tumors, similar to Kadcyla.

## Introduction

Cancer therapies have significantly improved in recent years due to the development of antibody-based therapeutics that confer high selectivity for either direct tumor targeting [1], or for the stimulation of anti-tumor immunity [2]. In the area of direct tumor targeting, current interest is particularly strong for the drug class of Antibody Drug Conjugates (ADCs), due to the outstanding efficacies and the clinical success of recently FDA-approved ADCs brentuximab-vedotin (Adcetris) and ado-trastuzumab-emtansine (T-DM1, or Kadcyla) [3–5]. By delivering a toxic payload to target cells via specific antibody binding, ADCs follow the same therapeutic principle as immunotoxins, bacterially produced fusion proteins comprising antibody fragments and highly potent bacterial toxins [6]. However, due to the high immunogenicity of the bacterial toxins in humans, immunotoxins are typically associated with a significant immune response, limiting repeated treatment cycles [7]. Thus, despite long-standing interest and significant progress in limiting immunogenicity [8,9], no immunotoxin has been approved to date. In contrast, since ADCs typically consist largely of human sequences their immunogenicity is manageable [10,11], and numerous treatment cycles are typically possible. Given this specific advantage of ADCs and the commercial success of the two ADC approved for therapy, more than 30 ADCs are currently at various stages of clinical development [12,13].

ADCs are a challenging class of drugs, since for optimal therapeutic effect the small molecular weight toxic payload on one hand needs to be tightly coupled to the antibody, but on the other hand requires specific release upon binding to and internalization into cancer cells. All ADCs currently applied in the clinic, or in clinical trials, have been manufactured using chemical conjugation involving linkers that covalently attach the toxic payload to either primary amino groups of lysine residues in the antibody structure, or to free thiol groups that are usually generated by mild reduction of intra-chain disulphide bridges of the antibody [14,15]. Because antibodies contain many lysine and cysteine residues, this approach produces heterogeneous mixtures of drug substances that present challenges with respect to analytical characterization and manufacturing. Despite their average drug-to-antibody ratio (DAR) of approximately 3.5 to 4, currently approved ADCs comprise individual components with DARs ranging from 0 to 8 [14], each behaving differently with respect to their pharmacokinetic, efficacy, and safety profiles [16].

In addition to the inherent heterogeneity of ADCs generated by this standard chemical conjugation, the maleimide-based linkers used in all ADCs currently in clinical trials and on the market have been found to exhibit instability in human serum. The maleimide-linker reaction can be reversed by the free thiol group of cysteine-34 in human serum albumin by a retro-Michael reaction [17]. This leads to a certain level of premature and systemic release of toxic payload from the ADCs, before they have reached their cancer targets. This is likely to have a negative impact on the efficacy and side-effect profile of such maleimide linker-containing ADCs.

As a result of these short-comings of traditional chemical conjugation, there is currently great interest in the further development of drug conjugation technologies, not only on site-specific payload conjugation to generate homogeneous ADC drug substances [14,18], but also on novel linkers that avoid maleimide-based chemistries with suboptimal *in vivo* stability 19].

One possible solution to both problems with current ADC formats is the enzymatic conjugation of payloads [14,18], e.g. by using sortase enzymes. Originally identified as a therapeutic target in gram-positive bacteria [20,21], this class of enzymes, and particularly sortase A from *S*. *aureus*, has been recognized for some time as a useful protein engineering tool, allowing the ligation of oligo-glycine-containing polypeptides or small molecules to proteins containing a sortase-penta-peptide motif (LPXTG in case of *S*. *aureus* sortase A) [22–24].

The sortase enzyme catalyzes a transpeptidation reaction that has been characterized best for sortase A of *S*. *aureus* [25]. The transpeptidation is initiated by a nucleophilic attack of the thiol group of Cys-184 of sortase A to the peptide bond between the threonine and the glycine of the LPXTG penta-peptide motif, resulting in a covalent thioacyl intermediate and the release of the terminal glycine of the LPXTG penta-peptide motif, including additional C-terminal sequences, if present. This LPXT-thioacyl-enzyme intermediate can then be resolved by entry of the primary amino group of an oligo-glycine stretch acting as a nucleophile. This way, a new peptide bond is formed between the threonine and the incoming glycine, as the sortase enzyme is released as unmodified enzyme for the next catalytic cycle.

The sortase transpeptidation reaction was initially only explored for the ligation of polypeptides [26]. Later, its utility was also shown for the conjugation of oligo-glycine-modified non-protein molecules to LPETG-containing proteins [23,27]. Recently, this was further extended to the labeling of antibodies or antibody fragments with small molecule labels or protein moieties [28–31]. However, the use of sortase enzymes for the generation of ADCs involving full-length antibodies and highly potent small molecular weight toxins and an initial functional characterization versus chemically conjugated ADCs has not been described to date.

Here, we present an enzymatic conjugation platform called SMAC-technology (sortase-mediated antibody conjugation technology) that allows efficient generation of homogenous ADCs involving full-length antibodies with pre-defined drug-to-antibody ratios. We describe the generation and functional characterization of several ADCs with different payloads and linker structures, including *in vitro* and *in vivo* tumor killing experiments in comparison to benchmark ADCs generated by classical chemical conjugation. We show that the SMAC-technology is capable of producing homogeneous ADCs with *in vitro* and *in vivo* potencies similar to traditional ADCs used in the clinic.

## Methods

### Generation of recombinant antibodies

The IgH and IgL chain variable region sequences of monoclonal antibodies brentuximab (clone Ac10) and FRP5 were obtained from patents US2008213289A1 and EP0502812A1, respectively. Those of trastuzumab (clone 4D5) were obtained from the IMGT database (http://www.imgt.org/3Dstructure-DB/cgi/details.cgi?pdbcode=7637). The Ac10 and FRP5 mouse variable regions were fused to a generic, mouse IgH chain-derived N-terminal hydrophobic signal peptide. In the case of trastuzumab, the published signal peptide sequence was used. For expression as whole IgG_1_, the open reading frames encoding human gamma-1 IgH and human kappa IgL chain constant regions were designed, each either untagged or C-terminally tagged with a sortase A recognition sequence and a Strep II affinity purification tag. The sequence appended to the C-terminus of the IgH chains was LPETGGWSHPQFEK, the sequence variants appended to the C-terminus of the IgL chain were G_1–4_SLPETGGWSHPQFEK. Gene synthesis of DNA fragments encoding each of these variable and constant regions, flanked by suitable restriction sites, was performed by GenScript (Piscataway, USA). Expression constructs encoding each of the full-length IgH and IgL chains were assembled in the proprietary mammalian expression vector pEvi5 by Evitria (Schlieren, Switzerland) and verified by DNA sequencing. Expression of the different monoclonal antibodies was performed in suspension-adapted CHO K1 cells by Evitria (Schlieren, Switzerland). Supernatants from pools of transfected CHO K1 cells were harvested by centrifugation and sterile filtered (0.2 μm) before FPLC-based affinity purification using Amsphere protein A columns (JSR Life Sciences). Antibodies were eluted from the protein A resin in 0.1M glycine pH 2.5 to 3.5 and immediately neutralized in 1M Tris-HCl buffer at pH 7.5. The eluted antibodies were either dialyzed overnight against Dulbecco's PBS, or the buffer was exchanged against Dulbecco's PBS by ultrafiltration.

### Synthesis of penta-glycine modified small molecules

Synthesis of Gly_5_-modified Fluorescein isothiocyanate (FITC) was performed by Bachem (Bubendorf, Switzerland). Synthesis of Gly_5_-modified vcPAB-MMAE, DM1, and maytansine was performed by Concortis (San Diego, USA).

### Production of recombinant sortase A from *Staphylococcus aureus*

A synthetic gene encoding amino acids 60 to 206 of a highly active evolved sortase A was designed based on Genbank entry AF162687.1 and published information [32], C-terminally tagged with a hexa-histidine tag coding sequence, and produced by total gene synthesis (GenScript, Piscataway, USA). Bacterial expression and purification by Ni-NTA affinity chromatography was done as described [25,32].

### Sortase-mediated FITC conjugation

**Fig 1:**
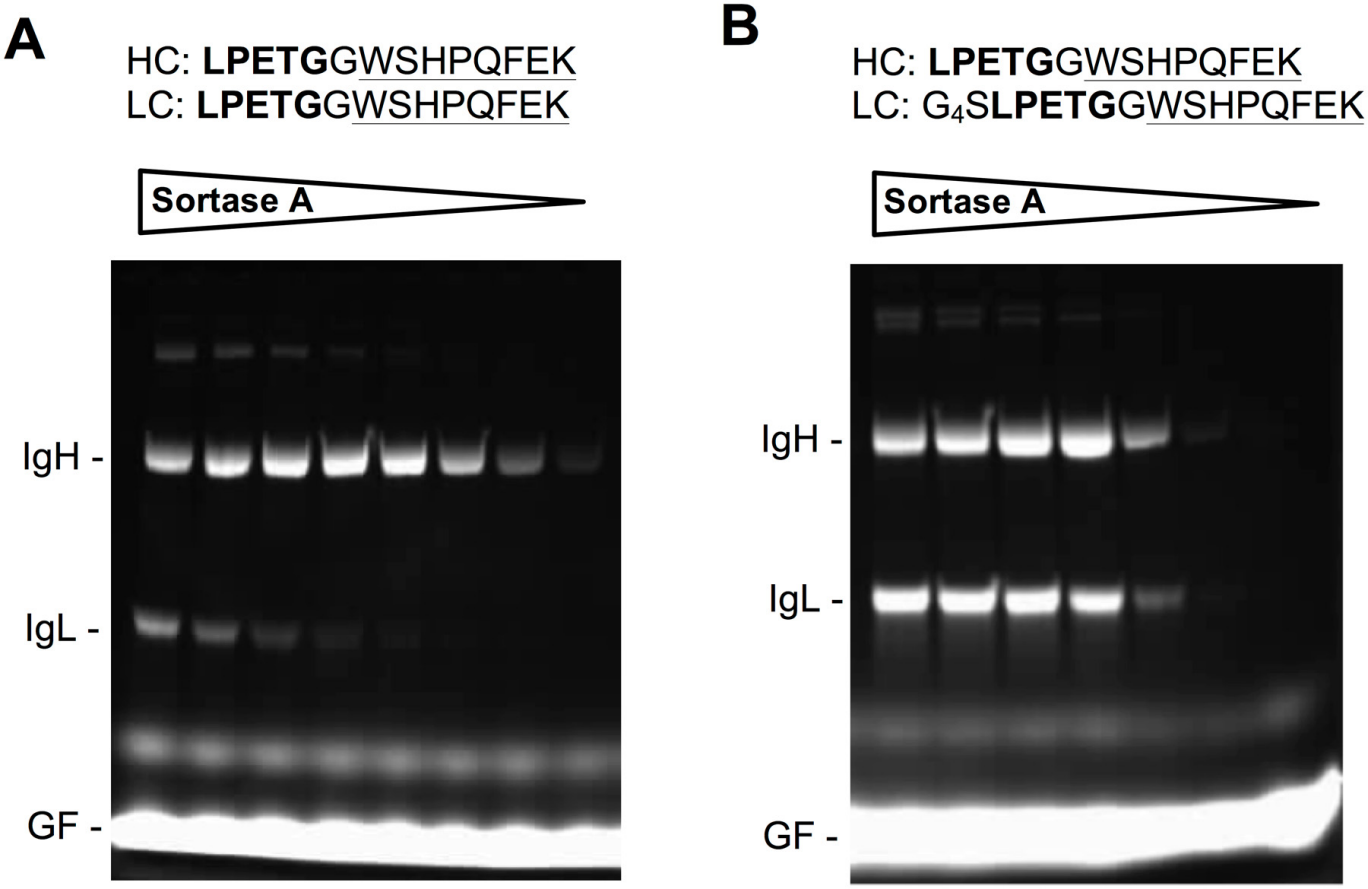
Sortase A-mediated conjugation of Gly_5_-modified FITC to C-terminally tagged mAb Ac10 IgH and IgL chains. The tag sequences are shown on top, with the sortase A recognition motif in bold print, and the Strep II tag underlined. (A) Spacer-free design; (B) Design using a 5 amino acid (GGGGS) spacer on the IgL chain. Antibody was incubated with 2-fold serial dilutions of sortase A in the presence of a 20-fold excess of Gly_5_-modified FITC. The crude reaction products were then separated by SDS-PAGE, and the FITC conjugates visualized by placing the gel on a UV box. IgH, heavy chain; IgL, light chain; GF, Gly_5_-FITC.

Conjugation efficiency to the C-terminus of antibody IgH and IgL chains was determined using Gly_5_-modified FITC as substrate. For this, monoclonal antibody [5μM] was incubated with Gly_5_-FITC [100μM] in the presence of 2-fold serial dilutions of sortase A (starting concentration Fig 1A, 5μM; Fig 1B, 2.5μM) in 25mM Tris-HCl, 150mM NaCl, 7.5mM CaCl_2_, pH 8.2 for 4h at 42°C. The reactions were stopped by addition of reducing SDS-PAGE loading buffer followed by a 5 minute incubation at 99°C. Reaction products were separated by size on an 8–16% gradient SDS-PAGE gel and visualized by placing the gel on a UV box.

### Sortase-mediated toxin conjugation

Toxins were conjugated to mAbs by incubating LPETG-tagged monoclonal antibodies [10μM] with Gly_5_-modified toxin [200μM] in the presence of 0.62μM sortase A in 50mM Hepes, 150mM NaCl, 5mM CaCl_2_, pH 7.5 for 3.5h at 25°C. The reaction was stopped by passing it through a Protein A HiTrap column (GE Healthcare) equilibrated with 25mM sodium phosphate pH 7.5, followed by washing with 5 column volumes (CVs) of buffer. Bound conjugate was eluted with 5 CVs of elution buffer (0.1M succinic acid, pH 2.8) with 1 CV fractions collected into tubes containing 25% v/v 1M Tris-base to neutralize the solution. Protein containing fractions were pooled and formulated in 10mM sodium succinate pH 5.0, 100mg/mL trehalose, 0.1% w/v polysorbate 20 by G25 column chromatography using NAP-25 columns (GE Healthcare) according to the manufacturer’s instructions.

### ADC analytics

The aggregate content of each conjugate was assessed by chromatography on a TOSOH TSKgel G3000SWXL 7.8mm x 30cm, 5μm column run at 0.5mL/min in 10% IPA, 0.2M potassium phosphate, 0.25M potassium chloride, pH 6.95. The drug loading was assessed by both Hydrophobic Interaction Chromatography (HIC) and Reverse Phase Chromatography (RP-C). HIC was performed on a TOSOH Butyl-NPR 4.6mm x 3.5cm, 2.5μm column run at 0.8mL/min with a 12 minute linear gradient between A—1.5M (NH_4_)_2_SO_4_, 25mM NaPi, pH = 6.95±0.05 and B—75% 25mM NaPi, pH = 6.95±0.05, 25% IPA. Reverse phase chromatography was performed on a Polymer Labs PLRP 2.1mm x 5cm, 5μm column run at 1mL/min/80°C with a 25 minute linear gradient between 0.05% TFA/H_2_O and 0.04% TFA/CH_3_CN. Samples were first reduced by incubation with DTT at pH 8.0 at 37°C for 15 minutes.

### *In vitro* cytotoxicity assays

Cytotoxicity of anti-HER-2 ADCs was investigated using SKBR3 cells, a human breast cancer cell line overexpressing HER-2, and T47D cells, a breast cancer cell line naturally expressing low levels of HER-2. Cytotoxicity of anti-CD30 ADCs was investigated using Karpas-299, a non-Hodgkin’s lymphoma cell line expressing high levels of CD30, and L428, a Hodgkin’s lymphoma cell line expressing low to moderate levels of CD30. Cells were plated on 96 well plates in 75μl growth medium (breast cancer cells: DMEM/10%FCS; lymphoma cells: RPMI/10% FCS) at a density of 10,000 cells per well and grown at 37°C in a humidified incubator in a 5% CO_2_ atmosphere. After one day, 25μl of 3.5-fold serial dilutions of each ADC in growth medium were added, resulting in final ADC concentrations ranging from 20μg/ml to 0.25ng/ml. Each dilution was done in duplicate. After 3 to 4 additional days, plates were removed from the incubator and equilibrated to room temperature. After approximately 30 minutes, 100μl CellTiter-Glo Luminescent Solution (Promega, Cat.No G7570) was added to each well and, after shaking the plates at 450rpm for 5 min followed by a 10 min incubation without shaking, luminescence was measured on a Tecan Infinity F200 with an integration time of 1 second per well.

### *In vivo* experiments

5x10^6^ SKOV-3 tumor cells (ATCC, HTB-77) in 200μl PBS/Matrigel (1:1) were implanted subcutaneously into the left flanks of NMRI nude mice. In the following, primary tumor volumes were measured by calipering. 29 days later, when a mean tumor volume of approximately 100–200mm^3^ was reached, tumor-bearing animals were randomized into groups of 8 animals each according to tumor sizes. Animals were treated on the same day (day 0, i.e. day of randomization) and 21 days later by intravenous injection of the respective ADC (15mg/kg), trastuzumab (15mg/kg) or vehicle control. Tumor sizes were monitored for a total of 43 days, after which the study was terminated, all animals sacrificed and a necropsy performed. Primary tumor tissue was collected from all animals and primary tumor wet weights were determined.

### Ethics statement

Animal experiments have been approved by the Ethics Committee for Animal Experimentation of the regional board Freiburg, Germany. The experimental protocol was registered by the regional board Freiburg, Germany (permit number G-13/23, reference number 35–9185.81/G-13/23). During the whole course of animal experiments, all efforts were made to minimize suffering.

## Results

### Sortase A-mediated conjugation to antibody IgH and IgL chains

**Fig 2:**
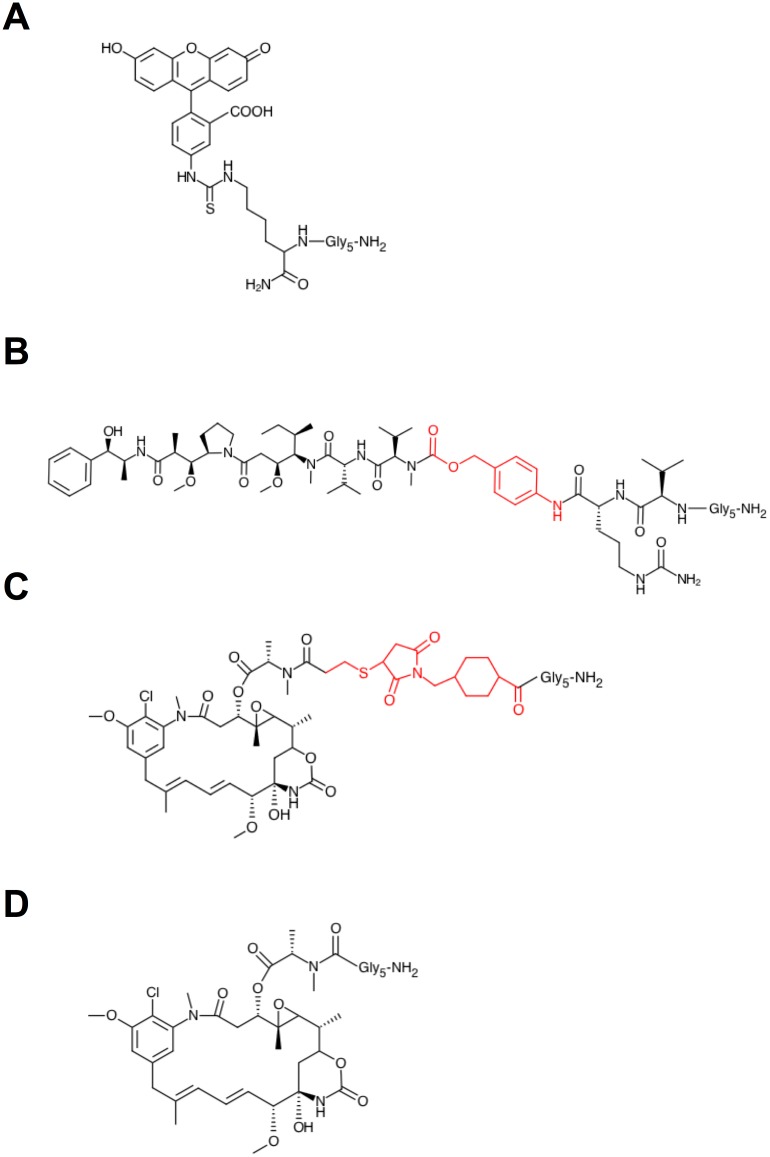
Structures of Gly_5_-modified small molecules used in this study. (A) Gly_5_-FITC; (B) Gly_5_-vcPAB-MMAE, with the cleavable vcPAB linker used in Adcetris shown in red; (C) Gly_5_-DM1, with the non-cleavable SMCC linker used in Kadcyla shown in red; (D) Gly_5_-maytansine, with the toxin directly coupled to the Gly_5_-pentapeptide. In each structure the free N-terminus of the penta-peptide tag is written out as an-NH_2_ amino group, in order to provide information about the orientation of the peptide tag.

The feasibility and efficiency of small molecule conjugation to antibody IgH and IgL chains was first evaluated using fluorescein isothiocyanate (FITC) as a substrate. To this end, the CD30-specific chimeric mAb cAc10 [33], principal component of the marketed ADC brentuximab vedotin (Adcetris) [4], was produced with each of its C-termini tagged with a sortase A recognition motif, and a Strep II affinity purification tag [34]. Since successful sortase A-mediated conjugation leads to a loss of the Strep II tag, this design allows for removal of unreacted substrate by Strep-Tactin affinity chromatograpy. The sequence thus appended to each of the C-termini was LPETGGWSHPQFEK. Further, for sortase A-mediated conjugation, the FITC molecule was modified by attachment of a penta-glycine via a lysine linker, resulting in Gly_5_-FITC (Fig 2A). Through this modification, the Gly_5_-FITC can serve a substrate for sortase A transpeptidation reactions.

A functionally enhanced evolved sortase A (eSrtA) [32] was produced as a recombinant protein *in E*.*coli* and purified by a one-step Ni-NTA affinity purification, as described before [25]. In order to evaluate the relative efficiency of conjugation to IgH and IgL chains, serial dilutions of eSrtA were tested for conjugation of Gly_5_-FITC to LPETG/Strep II-tagged mAb cAc10. Gly_5_-FITC covalently coupled to the IgH and IgL chains was visualized by UV-irradiation after denaturing SDS-PAGE of the conjugated cAc10 antibody. If the LPETG/StrepII-tag was directly appended to the C-termini of IgH and IgL chains, conjugation to the IgH chain was found to be highly efficient, and the enzyme could be diluted to a concentration 16-fold below that of the mAb without apparent loss of conjugation efficiency (Fig 1A). This corresponds to a 64-fold molar excess of C-termini over enzyme and clearly demonstrates the enzymatic processivity of eSrtA. Significantly, eSrtA-mediated conjugation to the IgL chain was substantially less efficient than to the IgH chain. Even when using eSrtA and mAb at equimolar concentration, only incomplete labeling was detected (Fig 1A). Since the human kappa IgL chain comprises a C-terminal cystein-residue involved in an interchain disulfide bond, it is conceivable that the LPETG sortase A recognition motif at IgL chains is not as accessible for the sortase enzyme and the substrate due to steric hindrance, which is one possibile explanation of the inefficient sortase-mediated conjugation at IgL chains.

In order to test this hypothesis, we decided to introduce a short peptide spacer between the C-terminus of the IgL chain and the sortase A recognition motif, with the assumption that this may improve conjugation efficiency. Thus, several LPETG/Strep II-tagged mAb cAc10 variants were produced, with identical IgH chains to those described, but comprising a 2 to 5 amino acid linker between the C-terminus of the IgL chain and the LPETG/Strep II tag (tag sequence: G_1–4_SLPETGGWSHPQFEK). Serial dilutions of eSrtA were then used to conjugate Gly_5_-FITC to the four different cAc10 variants. As expected, conjugation efficiency to the IgH chain remained unaffected and was equally efficient in all four antibody variants (data not shown). In contrast, conjugation to the IgL chain was progressively improved by increasing peptide-spacer length (data not shown). Significantly, with the longest peptide-spacer analyzed (GGGGS), conjugation of the Gly_5_-FITC substrate to the IgL chain was equally efficient as to the IgH chain (Fig 1B). Antibodies tagged in this manner were used throughout the rest of this study.

To investigate whether Sortase-mediated conjugation of small molecule payloads interferes with antigen binding, FITC-conjugated mAb cAc10 was used to stain CD30-expressing Hodgkin's lymphoma cells. As expected from the location of the conjugation site on the C-termini of IgH and IgL chains, *i*.*e*. most distal from the antigen binding sites, conjugation of FITC had no adverse effect on antibody binding (data not shown).

### Generation and evaluation of a site-specifically conjugated cAc10 (brentuximab)-MMAE conjugate

As a first proof-of-concept study, an eSrtA-conjugated version of the CD30-specific ADC brentuximab vedotin (Adcetris), currently marketed for the treatment of Hodgkin's lymphoma, was produced. Brentuximab vedotin contains the synthetic dolastatin-10 analog MMAE, chemically linked to mAb cAc10 via a cleavable linker, consisting of the protease-sensitive dipeptide valine-citrulline (vc) and a self-immolative spacer, *p*-aminobenzylcarbamate (PAB). For eSrtA-mediated conjugation, vcPAB-MMAE was modified by the addition of a penta-glycine to the valine-citrulline di-peptide, resulting in the Gly_5_-vcPAB-MMAE substrate (Fig 2B). The LPETG/StrepII-tagged mAb cAc10 was conjugated to Gly_5_-vcPAB-MMAE and purified by a single-step protein A affinity chromatography. The resulting ADC was analyzed by hydrophobic interaction chromatography (HIC) and reverse phase chromatography (RP) to determine the drug load, and by size exclusion chromatography (SEC) to determine the monomer content (Table 1). The DAR was determined to be approximately 3.2, implying an eSrtA conjugation efficiency per attachment site of roughly 80% under the non-optimized reaction conditions employed. The ADC had a slightly higher aggregate content when compared to the naked antibody, but with approximately 96% monomer was of acceptable quality for *in vitro* assays.

**Table 1:** Analytical summary of conjugates manufactured in this study.

Conjugate	Target	DAR	Mono (start)	Mono (conj)
cAc10-vc-PAB-MMAE	CD30	3.18	100	95.8
Trastuzumab-DM1	HER-2	3.05	98.1	98.1
Trastuzumab-maytansine	HER-2	3.28	98.1	97.6
cFRP5-DM1	HER-2	3.44	98.5	97.4
cFRP5-maytansine	HER-2	3.53	98.5	97.5

DAR, drug-to-antibody ratio, determined by hydrophobic interaction and/or reverse phase chromatography; Mono (start/conj), % momomer content before/after conjugation, determined by size exclusion chromatography.

**Fig 3:**
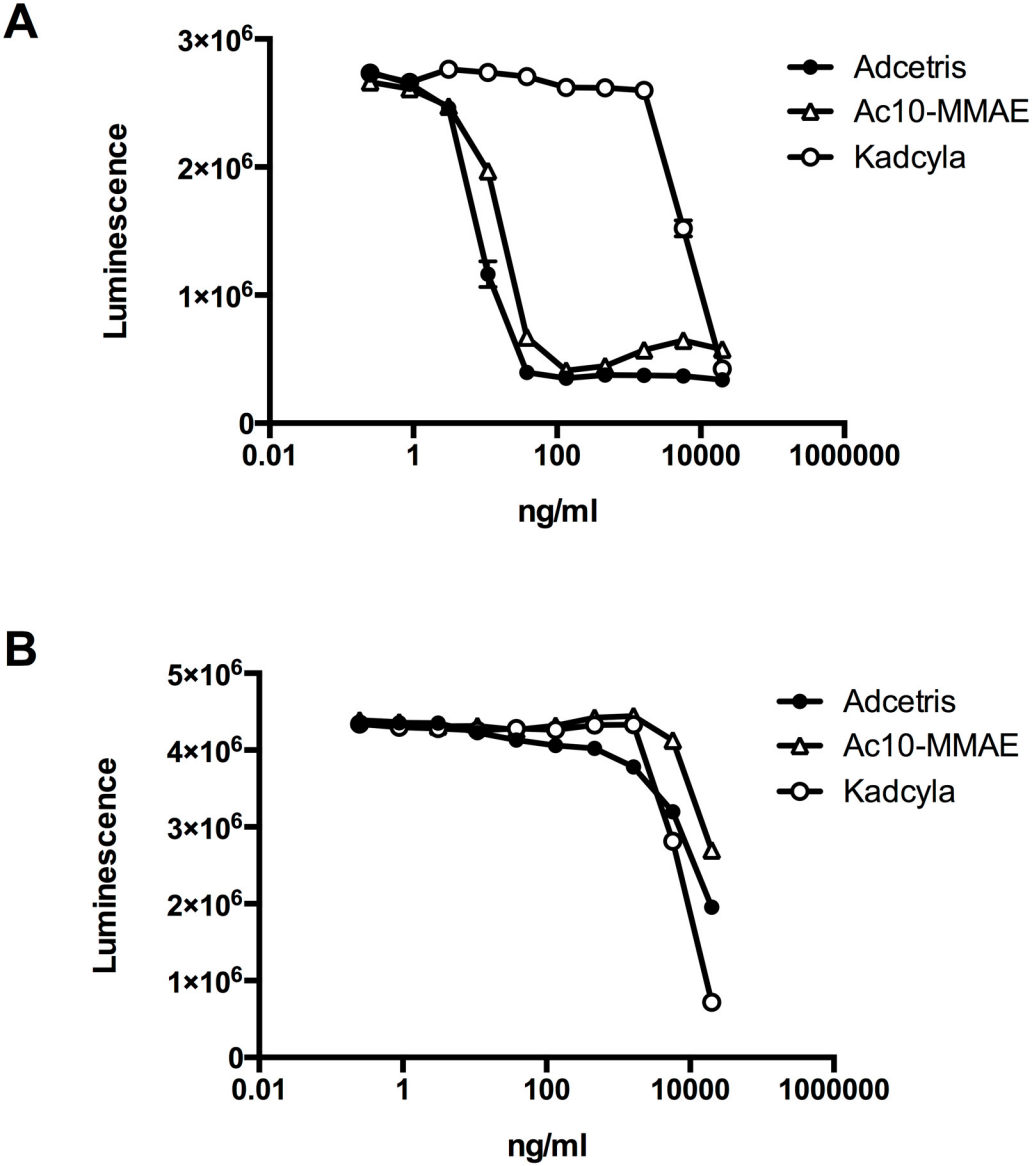
*In vitro* killing of CD30-positive Hodgkin’s and non-Hodgkin’s lymphoma cells by sortase A-conjugated anti-CD30 ADCs. CD30^high^ Karpas-299 non-Hodgkin’s lymphoma cells (A) and CD30^low^ L428 Hodgkin's lymphoma cells (B) were grown in the presence of serial dilutions of the indicated ADCs. Viable cells were quantified using a Luminescent Cell Viability Assay. Datapoints represent mean of two replicates and error bars represent SD.

Activity of the eSrtA-conjugated cAc10-vcPAB-MMAE was evaluated and compared to commercially available Adcetris in cell killing assays, using the CD30^HI^ non-Hodgkin's lymphoma (NHL) cell line Karpas-299 and the CD30^LO^ Hodgkin's lymphoma (HL) cell line L428 (Fig 3). Cells were plated in 96-well plates and either left untreated, or exposed to serial dilutions of each ADC, starting at a concentration of 20μg/ml. Kadcyla, which was used as a negative control, only had a moderate growth-inhibitory effect on either cell line at very high concentrations. In contrast, Adcetris and eSrtA-conjugated cAc10-vcPAB-MMAE potently killed the CD30^HI^ Karpas-299 cells, with IC_50_ values of 6.6ng/ml (≈44pM) and 12.2ng/ml (≈81pM), respectively, in the experiment presented (Fig 3A). In repeated independent experiments (six for Adcetris, and seven for eSrtA-conjugated cAc10-vcPAB-MMAE), the mean IC_50_ values and standard deviation were: Adcetris: 10.2ng/ml +/- 4.3ng/ml; eSrtA-conjugated cAc10-vcPAB-MMAE: 11.8ng/ml +/- 2.4ng/ml. Significantly, only very high concentrations of the two ADCs were able to kill the CD30^LO^ L428 cells, indicating that the toxicity of the ADCs is indeed specific and mediated by CD30 binding (Fig 3B). Thus, eSrtA-mediated conjugation yielded an ADC with *in vitro* properties similar to those of a traditional, chemically conjugated ADC.

### Generation and evaluation of HER-2-specific maytansinoid-conjugates

As an additional proof-of-concept, eSrtA-conjugated versions of the HER-2-specific ADC trastuzumab emtansine (T-DM1; Kadcyla), currently marketed for the treatment of breast carcinoma, was produced. Trastuzumab emtansine contains the maytansinoid DM1 [5], a cytotoxic agent binding to tubulin, chemically linked to the antibody via the non-cleavable linker Succinimidyl-4-(*N*-maleimidomethyl) cyclohexane-1-carboxylate (SMCC, designated MCC after conjugation). For eSrtA-mediated conjugation, two penta-glycine modified maytansinoids were produced. In one variant, the overall linker-drug structure of MCC-DM1 was maintained and the penta-glycine was attached to the linker, resulting in Gly_5_-DM1 (Fig 2C). In a second variant, the linker was omitted and the penta-glycine was directly attached to maytansine, resulting in Gly_5_-maytansine (Fig 2D). LPETG/Strep II-tagged trastuzumab was conjugated to Gly_5_-DM1 and Gly_5_-maytansine and purified by a single-step protein A affinity chromatography. To demonstrate universal applicability of eSrtA-mediated conjugation for the manufacturing of ADCs, also the mAb cFRP5, a chimeric version of the mouse mAb FRP5 [35], was used as a basis of HER-2-specific maytansinoid conjugates. Therefore, an LPETG/Strep II-tagged cFRP5 was similarly conjugated to Gly_5_-DM1 and Gly_5_-maytansine and purified by a single-step protein A affinity chromatography. The resulting ADCs were analyzed by HIC, RP and SEC to determine drug load and monomer content (Table 1). The DARs were determined to be between 3.05 and 3.53, suggesting an eSrtA conjugation efficiency per attachment site ranging between 76% and 88% under the reaction conditions employed. The aggregate contents of the ADCs were comparable to the respective unconjugated antibody.

**Fig 4:**
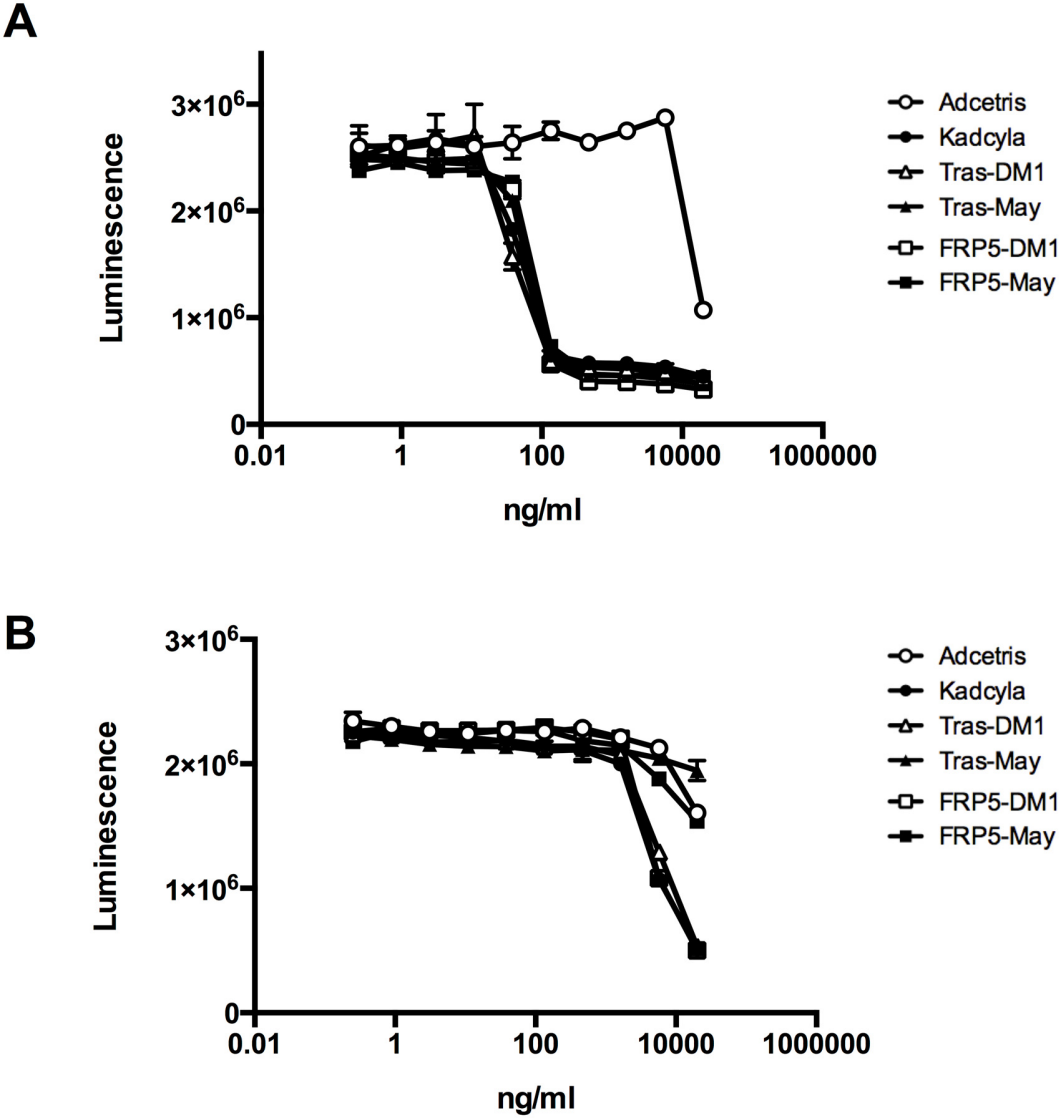
*In vitro* killing of HER-2-overexpressing breast cancer cells by sortase A-conjugated anti-HER-2 ADCs. HER-2-overexpressing SKBR3 cells (A) and HER-2-non-overexpressing T47D cells (B) were grown in the presence of serial dilutions of the indicated ADCs. Viable cells were quantified using a Luminescent Cell Viability Assay. Datapoints represent mean of two replicates and error bars represent SD.

The potencies for tumor cell killing of the eSrtA-conjugated maytansinoid-based ADCs were evaluated and compared to commercially available Kadcyla using the HER-2 overexpressing breast cancer cell line SKBR3 and, as a control, the breast cancer cell line T47D expressing low to moderate levels of HER-2 (Fig 4). Cells were plated in 96-well plates and exposed to serial dilutions of each ADC, starting at a concentration of 20μg/ml. Adcetris, which was used as a negative control, only had a moderate growth-inhibitory effect on either breast cancer cell line at very high concentrations. In contrast, Kadcyla and the eSrtA-conjugated ADCs trastuzumab-DM1, trastuzumab-maytansine, cFRP5-DM1, and cFRP5-maytansine each potently killed the HER-2-overexpressing SKBR3 cells, with comparable IC_50_ values of 48.4ng/ml (≈323pM), 40.5ng/ml (≈270pM), 62.9ng/ml (≈419pM), 67.0ng/ml (≈447pM), and 77.9ng/ml (≈519pM), respectively (Fig 4A). In eight independent experiments, the mean IC50 of Kadcyla and trastuzumab-maytansine was quite consistent and determined to be 27.7ng/ml +/- 12.7ng/ml SD and 29.9ng/ml +/- 14.4ng/ml SD, respectively. As expected, only very high concentrations of the five ADCs were able to kill the HER-2^LO^ T47D cells, indicating that the toxicity of the ADCs is indeed specific and mediated by HER-2 binding (Fig 4B). Therefore, it was concluded that eSrtA-mediated conjugation yields ADCs with *in vitro* cell killing potencies similar to those of a traditional, chemically conjugated ADC. In addition, the similar *in vitro* potency is obtained regardless of whether an MCC linker structure is still contained in the payload, or whether the maytansine payload is directly conjugated to the antibody via a Gly5-containing peptide linker attached to the core structure of the toxin.

### *In vivo* anti-tumor activity of HER-2-specific maytansinoid-conjugates

Next, the efficacy of the trastuzumab- and cFRP5-based ADCs was evaluated *in vivo* in a mouse model of HER-2-overexpressing ovarian cancer. Given the comparable *in vitro* potencies of eSrtA-conjugated DM1- and maytansine-based ADCs, it was concluded that the presence of the MCC linker was dispensable. Therefore, it was decided to only test eSrtA-conjugated trastuzumab-maytansine and cFRP5-maytansine ADCs *in vivo* and to compare them to commercially available Kadcyla. To better understand the contribution of the toxic payload of the antibodies to *in vivo* anti-tumor activity, unconjugated trastuzumab [36], a therapeutic antibody marketed as Herceptin for the treatment of certain forms of breast and metastatic gastric cancer [37–39], was also included in the study.

**Fig 5:**
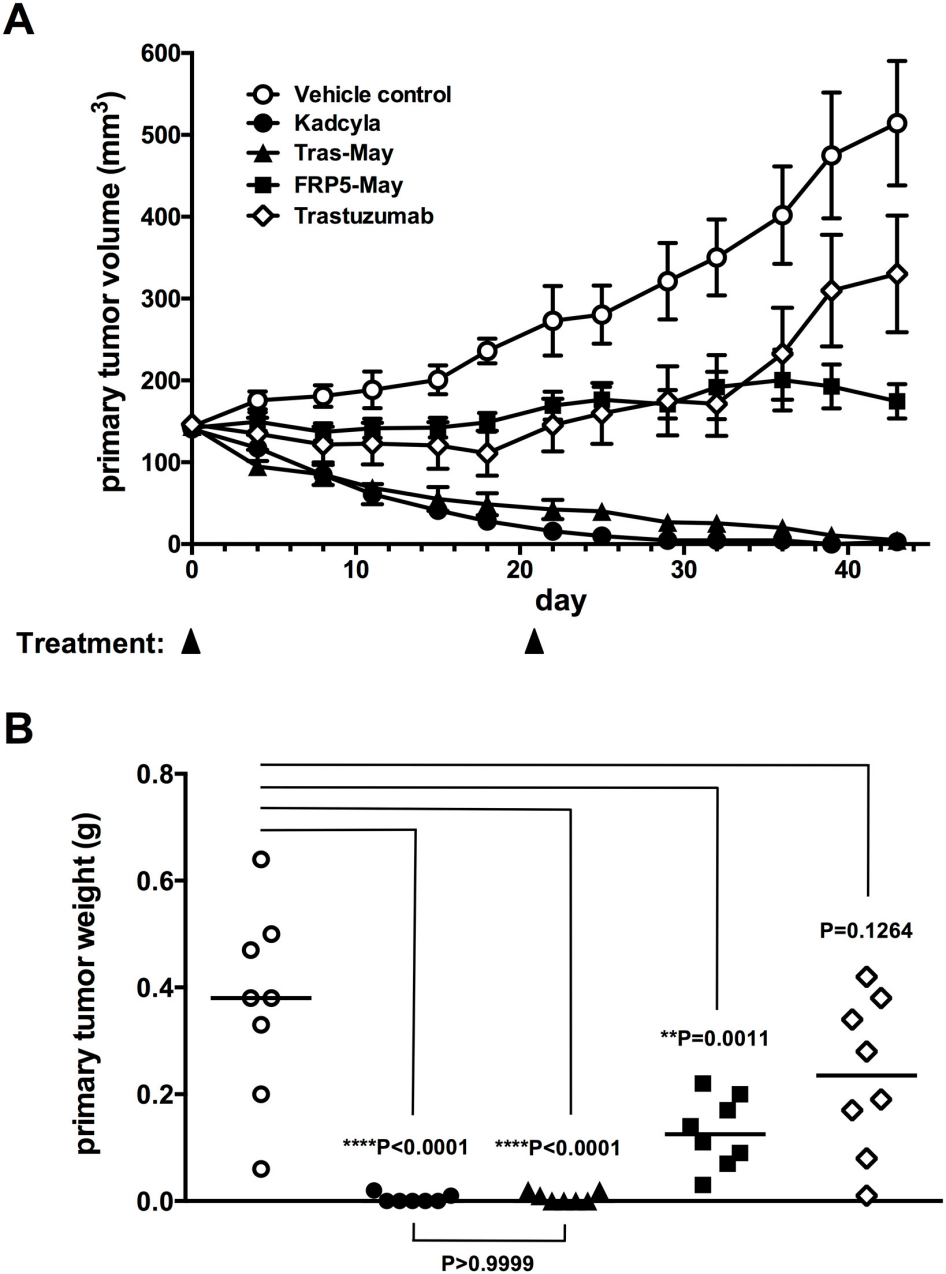
*In vivo* evaluation of HER-2-specific ADCs in a mouse xenograft model. SKOV3 human ovarian carcinoma cells were grown subcutaneously in nude mice. Animals were treated i.v. with the indicated ADC (15mg/kg), trastuzumab (15mg/kg) or vehicle control on days 0 and 21. (A) *In vivo* monitoring of tumor growth until day 43. Data points represent mean and bars represent SEM. (B) Tumor weights determined at necropsy on day 43. Statistical analysis was done with GraphPad Prism 6 (GraphPad Software, Inc., San Diego, USA) using one-way ANOVA with the Tukey's post hoc test. Asterisks indicate level of significance.

As a validated mouse model for HER-2 positive cancer, the human SKOV3 ovarian cancer cell model, expressing approximately 2.6x 10^5^ molecules of HER2 per cell [40], was employed. For this, SKOV3 cells were implanted subcutaneously into NMRI nude mice and allowed to grow to an average size of approximately 150 mm^3^. Mice were then given i.v. injections of 15mg/kg of each ADC or trastuzumab on days 0 and 21 and tumor development was monitored until day 43, after which the study was terminated (Fig 5). Tumors in vehicle control mice grew to an average size of over 500mm^3^ during this period of time. Treatment with unconjugated trastuzumab led to inhibition of tumor growth during the first few weeks, after which the tumors continued to grow (Fig 5A). In contrast, in animals treated with either the eSrtA-conjugated trastuzumab-maytansine ADC or the commercial Kadcyla ADC, the tumors continuously regressed during treatment (Fig 5A) and were essentially undetectable at the end of the study (Fig 5B). Significantly, although the cFRP5-maytansine conjugate displayed comparable potency in *in vitro* killing assays, it did not cause tumor regression like the ADCs based on trastuzumab, but only lead to stabilization of the tumor during the entire time of the study (Fig 5A). Thus, although the cFRP-5 maytansine ADC displayed higher efficacy than unconjugated trastuzumab, it was less efficacious than the trastuzumab-based ADCs. This mAb-dependent difference in ADC efficacy is not unexpected and may be associated with differences in binding properties and internalization rates, which were not investigated further. Taken together, eSrtA-mediated conjugation yielded a novel trastuzumab-maytansine ADC with similar *in vitro* and *in vivo* potency for tumor cell killing as the benchmark ADC Kadcyla.

## Discussion

Sortase-enzyme mediated conjugation of small molecular weight toxic payloads to full-length antibodies represents a novel approach for the development of site-specifically conjugated ADCs. While it was previously known that sortase enzymes can be exploited to conjugate small molecules modified with glycine residues to protein substrates containing a sortase recognition motif [23], application of this approach for the efficient generation ADCs, which exhibit satisfactory potency for tumor cell killing, has not yet been demonstrated.

Here we show that penta-glycine modification of maytansine and MMAE, the most common toxic payloads used in ADCs currently in clinical development, allows efficient sortase A-mediated conjugation to LPETG-tagged antibodies. While conjugation to the IgL chain initially occurred with very poor efficiency, introduction of a 5 amino acid spacer between C-terminus and sortase recognition motif significantly increased efficiency. In such optimized "sortagged" mAbs, conjugation to IgL and IgH chains was equally efficient, allowing concomitant modification of all four sites. No further optimization of the sortase reaction was required and conjugation efficiencies of >80% were routinely achieved with 64-fold sub-stoichiometric quantities of sortase enzyme and 5-fold molar excess of glycine-modified payload substrate, respectively, per conjugation site. Significantly, similar conjugation efficiencies were observed with more than 10 different mAbs and several additional payloads, representing 2 different chemical backbones (data not shown). This indicates that sortase A-mediated conjugation is a generally applicable method to produce ADCs.

We have demonstrated that sortase-enzyme mediated conjugation of maytansine and MMAE payloads yields potent ADCs effecting efficient tumor cell killing. It is widely accepted that efficient release of toxins upon target binding and internalization requires functional linker structures, depending on the intracellular trafficking of the target-ADC complex into either early or late endosomal compartments [15]. Maytansine payloads are most frequently conjugated to antibodies with either cleavable N-succinimidyl-4-(2-pyridyldithio) butanoate (SPDB) or non-cleavable SMCC linker structures, which define them as DM4 or DM1 payloads, respectively [41]. The analysis of intracellular activation and the metabolites of DM1- and DM4-ADCs suggested that the non-cleavable DM1 payload is able to effect tumor killing upon complete hydrolysis of the ADC and release of the DM1 still attached to a lysine amino acid residue of the degraded antibody [41]. Therefore, we hypothesized that the pentaglycine-modified DM1 payload, conjugated via a peptide bond to the C-terminal sortase tag, might also be effective upon HER-2-dependent targeting, internalization and lysosomal degradation. The almost identical efficacies of chemically conjugated trastuzumab-DM1 and sortase enzyme-conjugated trastuzumab-DM1 for tumor killing *in vitro* do indeed suggest that the payloads are equally well released upon internalization and presumably lysosomal degradation. This could theoretically be explained by the presence of identical MCC structures in the chemically and enzymatically generated DM1 ADCs, which may result in identical or highly similar metabolites being released from the ADCs. In order to test whether the MCC linker structure is of functional importance, we have removed it from ADCs by utilization of a maytansine payload, in which the penta-glycine for sortase conjugation had been directly attached to the maytansine backbone by replacement of the methyl group of maytansine (see Fig 2D). It was not predictable whether such a minimal Gly_5_-maytansine payload conjugated via a presumably stable peptide bond to the C-termini of IgH and IgL chains would result in efficient release of a maytansine metabolite with sufficient potency. Analysis of the *in vitro* potencies of sortase-conjugated Gly_5_-maytanine containing ADCs indeed revealed that they were equally potent at killing HER-2-positive breast cancer cells as ADCs containing DM1 payloads. The comparable potency of chemically conjugated trastuzumab-DM1 and sortase-conjugated trastuzumab-maytansine ADCs *in vitro* was also confirmed by an *in vivo* xenograft mouse model, involving human HER-2-positive SKOV3 carcinoma cells. In mice treated with both chemically conjugated trastuzumab-DM1 (commercial Kadcyla) and sortase-conjugated trastuzumab-maytansine, complete tumor regression was observed in all mice of the respective treatment groups using the standard concentration of 15mg/kg employed in the majority of T-DM1 *in vivo* models. Therefore, it can be concluded that maytansine payloads can be used to generate highly potent ADCs by site-specific conjugation using sortase enzymes and involving the formation of peptide bonds in absence of any additional linker structures.

In case of the chemically conjugated anti-CD30 ADC brentuximab-vedotin (Adcetris), it has been published that full cell killing activity requires release of the payload inside tumor cells via cathepsin B mediated enzymatic cleavage at the valine-citrulline (vc) dipeptide linker, followed by release of free MMAE by self-immolation of the PAB-spacer [4]. Consistent with this, we have found that the sortase-conjugated anti-CD30-vcPAB-MMAE ADC was equally potent as the chemically conjugated ADC Adcetris, because the same cleavable linker structure was contained effecting efficient release of the MMAE payload. In further experiments, a trastuzumab-MMAE ADC was generated, in which the vcPAB linker was omitted by directly attaching the Gly_5_-linker to the MMAE toxin. Absence of the vcPAB moiety led to an ADC with very limited potency with an IC_50_ of >1**μ**g/ml on SKBR3 cells (data not shown). This is fully consistent with published data on conjugates with MMAE payloads [42]. In contrast, a sortase-conjugated trastuzumab-monomethylauristatin F (MMAF) ADC, in which the payload was directly attached to the Gly_5_ linker, effected efficient killing of SKBR3 cells with an IC_50_ similar to that of Kadcyla and the other maytansine-conjugates (data not shown). This observation is also consistent with previously published data, in which highly potent chemically conjugated MMAF ADCs have been generated without inclusion of a cleavable vcPAB linker structure [43].

In summary, it can be concluded that sortase-enzyme mediated conjugation of small molecule payloads can be used to efficiently generate site-specifically conjugated ADCs that exhibit potencies for tumor killing *in vitro* and *in vivo*, that are similar to chemically conjugated ADCs. These high potencies can be achieved by completely omitting complex linker structures that are traditionally contained in most ADCs currently in clinical testing, or already approved for patient treatment.
